# Supplementary material for: Reference bioimaging to assess the phenotypic trait diversity of bryophytes within the family Scapaniaceae
Source: Sci Data. 2022 Oct 4;9:598. doi: 10.1038/s41597-022-01691-x (PMC9532418; doi:10.1038/s41597-022-01691-x)
Supplement: Supplementary file 3 — Supplementary Information [file 41597_2022_1691_MOESM3_ESM.docx]

### Supplementary Information

**Supplementary Table 1:** List of voucher specimens and fresh samples used in this study. All voucher specimens have been investigated. The columns list the taxonomic species identifiers (*NCBI*, *GBIF*, or *Open Tree of Life* identifiers, if available), the text on the specimen sleeves (collector, date and text on the envelopes) and the voucher specimen identifiers (the first letters either indicate the *Index Herbariorum* institution code^57^, if available, or the name of private collection where the specimens were stored).

| **Species names** | **Taxonomy IDs** | **Reference of voucher specimens** | **Voucher identifiers** |
| --- | --- | --- | --- |
| *Diplophyllum albicans* (L.) Dumort. | NCBI:264775  GBIF:5286350  OTT:1055640 | Urmi, E. (1999): Schweiz, Kt. Ticino, Caviono: Valle Di S. Abbondio, 704.40/106.98, ca. 850 müM | Urmi-8371 |
| *Diplophyllum obtusatum* (R.M.Schust.) R.M.Schust. | NCBI:2212631  GBIF:5710146  OTT:3855725 | Urmi, E. (2017): Schweiz, Kt. Ticino, Chiasso: im Bosca Penz oberhalb Bresciano, 721.27/76.88, ca. 300 müM | Urmi-10695 |
| *Diplophyllum obtusifolium* (Hook.) Dumort. | NCBI:248354  GBIF:5710147  OTT:831945 | Urmi, E. (1988): Schweiz, Kt. Ticino, Cugnasco: Alpe di Ruscada oberhalb Corte di mezzo, 715.00/119.00, ca. 1550 müM | Urmi-4501 |
| *Diplophyllum taxifolium* (Wahlenb.) Dumort. | NCBI:248355  GBIF:5710140  OTT:831946 | Urmi, E. (1975): Italien, Ot. Crigiono Mesocco, oberhalb San Bernardina, am linken Seitenback der Moesa nordwestlich von Careida Sot, Landeskarte 734.34/147.91, Felsnische an der Seitenwand der kleinen Schlucht, ca. 1680 müM. | Urmi-1060 |
|  |  | Peters, K. (2020): Schweiz, in Erdloch unter Felsspalte, 46.083813428, 7.923810274, 2614m +- 5m | Peters-2020-21 |
| *Douinia ovata* (Dicks.) H.Buch | NCBI:248357  GBIF:2689235  OTT:831948 | Urmi, E. (1980): Spanien, Prov. Oviedo (Asturias), Cangas de Narcea: Monte de Muniellos bei Tablizas oberhalb Venta Nueva, v. Madrid: 3°0'20''W / 43°2'10''N, ca. 820 müM, an trockenen Felsen | Urmi-2056 |
| *Scapania aequiloba* (Schwägr.) Dumort. | NCBI:464313  GBIF:7696978  OTT:382426 | Urmi, E. (1976): Schweiz, Kt. Zürich: Hütten, Chrüzbrunnen am Höhronen-Nordhang, auf anstehendem Molasse-Sandstein im Schluchtwald, ca. 910 müM, Ost-Exposition | Urmi-1111 |
|  |  | Urmi, E. (2018): Schweiz, Kt. Uri, Spiringen (Enklave Urnerboden): unterhalb Waldrüti, 712.09/194.26, ca. 1340 müM, Straßenböschung, Kalkfelsen | Urmi-10772 |
| *Scapania apiculata* Spruce | NCBI:537844  GBIF:4277163  OTT:655684 | Hürlimann, H. (1983): Japan, an morschom, trockenem Stamm in Birkenwald beim 5-gome am Mt. Fuji, Japan, ca. 2200 müM | Z-000130732 |
| *Scapania aspera* M.Bernet & Bernet | NCBI:399155  GBIF:7435100  OTT:683885 | Bertram, J. (2007): Schweiz, SO, Seewen, SW Fulnau, E Risenberg, 613.845/253.87 680 müM, Eingang zu einer Gasse zwischen steilen Felswänden im Fagetum, Felsabsatz, auf kantig-rauhem Kalkgestein | Bertram-3283a |
| *Scapania brevicaulis* Taylor | GBIF:4277188  OTT:3855710 | Macurian, S.M. (1900) | Zürich-00000 |
| *Scapania calcicola* (Arnell & J.Perss.) Ingham | NCBI:537846  GBIF:4277184  OTT:655680 | Hürlimann, H. (1968): Schweiz, Schattiger Kalkfelsen im obersten Teil des Berghügels Ramstein bei Bretzol BL | Z-000129406 |
| *Scapania carinthiaca* var. *massalongi* J.B.Jack ex Lindb. | GBIF:8320028 | Meier, M. (2019): Schweiz, Kt. SZ, Gemeinde Sattel, Bärenfang, Zufluss zum Lauitobelbach, Koord: 692.912/213.421+-6m, Höhe: 1300 müM, Waldbach, vermodernder, ins Bachbett ragender Stamm einer Fichte oder Tanne | ZT-337051 |
|  |  | Høitomt, T. (2018): Norway: Oppland: Vestre Slidre, Mosåni, På dødved av gran langs elva, Host: Picea abies, UTM: 499542,6769666, Alt: 400m, 3.7.2018 | TRH-B108431 |
| *Scapania compacta* (Roth) Dumort. | NCBI:402640  GBIF:9148635  OTT:168110 | Urmi, E. (1980): Italien, Sardegna, Prov. di Nuoro, Airtzo oberhalb des Dorfes am Weg nach Badu Arala, Ret. ital.: 1517.8 E / 4423.4 N, ca. 1020 müM, bei einem Brunnen an Felsen (kalkarm) | Urmi-1951 |
| *Scapania crassiretis* Bryhn | NCBI:248358  GBIF:4277199  OTT:831941 | Hürlimann, H. (1947): Italien, auf der Nordseite der Talsperre von Caraglia GR | Hürlimann-H252 |
| *Scapania curta* (Mart.) Dumort. | NCBI:537847  GBIF:2689216  OTT:655678 | Culmann, P. (1998) | ZT-7/93-5 |
| *Scapania cuspiduligera* (Nees) Müll.Frib. | NCBI:537848  GBIF:8306353  OTT:655688 | Urmi, E. (1990): Schweiz, Kt. Wallis, Evolene: Comba Louva am linken Talhang, CH1903/LV03: 601.00 / 109.00, ca. 1860 müM, Nadelwald offene Stelle auf stellenweise kalkreichem Stein | Urmi-5345 |
|  |  | Peters, K. (2020): Schweiz, auf Erde, Hang, 46.037564627, 7.954650076, 2240m, +-5m | Peters-2020-09 |
| *Scapania degenii* Schiffn. ex Müll.Frib. | NCBI:537848  GBIF:8306353  OTT:655688 | Gustafsson, L. (1989): Jämtland, Undersåker par., Ristafallen, S-side inbetween rocks near the stream, below the fall | CP-CM30374 |
| *Scapania glaucocephala* (Taylor) Austin | NCBI:537849  GBIF:4277200  OTT:655686 | Schuster, R.M. (1956): USA, Decaying logs, shaded swamp along small stream, Sturgeon Bay in Wildwood State Park, Emmet Co., Michigan | CP-CM30377 |
|  |  | Høitomt, T. (2018): Norway: Oppland: Etnedal, Fjellselve, På låg av osp i elvekant, Host: Populus tremula, UTM-WGS84: 534192,6750148, Alt: 375 | TRH-B108428 |
| *Scapania gracilis* Lindb. | NCBI:264776  GBIF:5837556  OTT:844266 | Urmi, E. (1982): Spanien, Prov. Oviedo (Asturias), Ponga ca 2.5km oberhakb Sellaño, am Rio Ponga (rechte Seite) v. Madrid: 1°31'10''W, 43°13'55''N, ca 280 müM, sehr steiler felsiger Hang, Felsstufe, kalkarm | Urmi-2211 |
| *Scapania gymnostomophila* Kaal. | NCBI:537850  GBIF:4277196  OTT:133595 | Urmi, E. (2016): Schweiz, Kt. Grischun, Valsot am Strässchen nördl. v. Dorf Tschlin, 827.66/195.71, ca. 1550 müM, Wandrand an der Strasse, schieferige Kalkfelsen | Urmi-10478 |
|  |  | Peters, K. (2020): Schweiz, auf Erde, west-exponierter Hang, 46.039167304, 7.954680641, 2237m, +-10m | Peters-2020-16 |
| *Scapania helvetica* Gottsche | NCBI:537851  GBIF:7787988  OTT:133598 | Urmi, E. (1998): Schweiz, Kt. Wallis, Zwischbergen: oberhalb Bidemji im Laggintal, CH1903/LV03: 647.00/111.00, ca 2100 müM, felsiger Hang mit lockerem Grünerlen-Gebüsch, über Silikatfelsen auf Sand | Urmi-8131 |
|  |  | Schnyder, N. (2012): Schweiz, UR, Realp, Furkapass, S Passhöhe, See bei Pt. 2650, 675.98/157.24, 2650m, Schneeboden feuchte Erde | ZT-210488 |
| *Scapania hyperborea* Jørg. | NCBI:402139  GBIF:8336103  OTT:247549 | Hallingbäck, T. (2019): Province: Jämtland. Parish: Åre, Locality: Staån, myrvegetationen, Long: 12.27523, Lat: 63.59544 | Hallingbäck-50160 |
| *Scapania irrigua* (Nees) Nees | NCBI:537852  GBIF:2689217  OTT:133601 | Urmi, E. (1992): Schweiz, Kt. Uri, Wassen: oberhalb Kartigel im Meiental, 684/174, ca 1950 müM, grasiger Hang mit felsiger Rinne, feuchtes kalkarmes Gestein | Urmi-6100 |
|  |  | Urmi, E. (2016): Schweiz, Kt. Graubünden, Val Müstair Buffalora, westlich vom Ofenpass 816.79/168.69, ca. 2110 müM, Flachmoor, Torfmoosbult | Urmi-10576 |
| *Scapania irrigua* subsp. *irrigua* (Nees) Nees | GBIF:7420792 | Söderström, L. (1991): Sweden, Västerbotten, Sävar sn, Grisselögern, Bland stenar på stranden (Ruta i rikets nät: 20K67) | UME-78146 |
| *Scapania irrigua* subsp. *rufescens* (Loeske) R.M.Schust. | GBIF:8087753 | Sørensen, T. (1933): Liverpool Land: Cape Hope, Lat 70.28, Long: 220.25 W | CP-CM30429 |
| *Scapania kaurinii* Ryan | NCBI:537853  GBIF:4277164  OTT:133603 | Laine, T. (1966): Enontekiö Lapland, Enontekiö, Porojärvat, Ridnitsohka mountain, In alpine top area, on soil, 1280m above sea level | Z-026-1966 |
| *Scapania ligulifolia* R.M.Schust. | NCBI:1230444  GBIF:8288472  OTT:279012 | Frisvoll, A.A. (1973): Svalbard: Dickson Land, Nordfjorden NØ. Vestskråningen av Kongressfjellet litt S for toppen, 300-400 moh | TRH-74054 |
| *Scapania lingulata* H.Buch | NCBI:402641  GBIF:4277202  OTT:2063 | Buch, H. (1919): Finlandia Regio aboensis, Insula Hirvensalo, St. Marie, ad rupes | Zürich Buch-1919 |
| *Scapania mucronata* H.Buch | NCBI:537854  GBIF:4277191  OTT:133587 | Urmi, E. (1992): Schweiz, Kt. Uri, Realp: ob den Galenbödmen, 675.00 / 159.00; ca 2530 m ü. M. | Urmi-6291 |
|  |  | Schnyder, N. (2003): Schweiz, NE Fleurier, Pouetta Raisse, 536.6 / 193.2, 1010 m | Schnyder-47044 |
|  |  | Buch, H. (1922): Finland, Regio aboensis, Angelniemi | Zürich, Buch-1922 |
| *Scapania nemorea* (L.) Grolle | NCBI:41848  GBIF:2689220  OTT:851174 | Urmi, E. (1981): Schweiz, Kt. Obwalden, Giswil: Altibach-Tobel beim Kleinteil, 654.36 / 186.25; ca. 650 m ü M. | Urmi-2110 |
| *Scapania nimbosa* Taylor | NCBI:1133313  GBIF:8635654  OTT:1062948 | Wallace, E. (1955) | Zürich Wallace-1955 |
| *Scapania obcordata* (Berggr.) S.W.Arnell | NCBI:402140  GBIF:4277171  OTT:719916 | Zemp, F. (2019): Schweiz, Kanton: Be, Gemeinde: Innertkirchen, Sustenpass, Steinsee, Koord: 675.220/174.460, Höhe: 2050m, Standort: Bachalluvion | Zemp-337400 |
| *Scapania obscura* (Arnell & C.E.O.Jensen) Schiffn. | NCBI:1282292  GBIF:4277165  OTT:558046 | Urmi, E. (2019): Schweiz Kt. Wallis, Oberwald beim Totensee auf dem Grimselpass, CH1903/LV03: 669.03 / 156.76, ca. 2170 müM, kleines saures Flachmoor neben dem Bach, relativ grober Silikat-Sand | Urmi-10928 |
|  |  | Culmann, P. (1908): Schweiz, Wallis, Grimsel, prope lac. d. Todtensee, in paludosis juxta rivulum, hygrophila, 2150m | Zürich-0000 |
| *Scapania ornithopodioides* (With.) Waddell | NCBI:264777  GBIF:7512997  OTT:333110 | Macricar, S.M. (1898): | ZT-1898 |
| *Scapania paludicola* Loeske & Müll.Frib. | NCBI:209818  GBIF:4277131  OTT:245460 | Urmi, E. (1985): Schweiz, Kt. Obwalden, Engelberg Feldmoos auf Gerschni, 673.70/184.82, ca. 1230 müM, Moor, sehr nasser Torf | Urmi-3307 |
| *Scapania paludosa* (Müll.Frib.) Müll.Frib. | NCBI:537855  GBIF:4277179  OTT:133589 | Urmi, E. (2019): Schweiz, Kt. Wallis, Oberwald beim Totensee auf dem Gimselpass, CG1903/LV03: 668.72/156.92, ca 2170 müM, moorige Stelle an einem Bach, teils im Wasser stehend, Silikat-Sand | Urmi-10924 |
| *Scapania parvifolia* Warnst. | GBIF:4277175  OTT:3855712 | Marko Lewis, 12.7.1976, Abundand amongst rocks near snow beds. Upper Ambler river, ridge above Kowalskis Creek, Mt. 3950, 67°19'N, 156°59'W. Late snow bed on N. side of alpine ridge, wet or moise, 2000 ft | CP-CM30382 |
| *Scapania praetervisa* Meyl. | GBIF:4277193  OTT:7571327 | Urmi, E. (1982): Schweiz, Kt. Grischun, Lumbrein hart an der Gemeindegrenze oberhalb Schlareins, CH1903/LV03: 729.14 / 173.20, ca. 1750 müM, am Anstehenden (schattig), kalkarmes schieferiges Gestein mit Ausblühungen | Urmi-2161 |
| *Scapania scandica* (Arnell & H.Buch) Macvicar | NCBI:1133317  GBIF:4277166  OTT:1062956 | Düll R. (1971): Schweiz, Kt. Uri, 8 km nördlich d. St. Gotthartpasses nahe d. Str. Grundgeb., ca 1570m | ZT-017155 |
|  |  | Urmi, E. (1999): Schweiz Kt. Ticino, Caviano Valle de S. Abbondio, CH1903/LV03: 704.40/106.98, ca. 850 müM, grasiger Felshang mit wenig Gebüsch, anstehender Silikatfels, z.T. mit etwas Feinmaterial | Urmi-8376 |
| *Scapania simmonsii* Bryhn & Kaal. | NCBI:537857  GBIF:4277140  OTT:133593 | Lewis, M. (1976): Upper Ambler River, 0.5 miles N. of Ulaneak Creek, base of Grey Mt. Tocky limestone slope | CP-CM30391 |
|  |  | Kjeld Holmen, 15.7.1961, Alaska: Old John Lake, Lat. 68.08N, Long. 145 W, Alt 0m CM-30384 |  |
| *Scapania sphaerifera* H.Buch & Tuom. | NCBI:537858  GBIF:5793125  OTT:133605 | Bakalin, V. (2010): Russian Far East. Primorsky Territory. Shkotovsky, District, Pidan Mt., N-facing slope in upper course of Oyry Stream. Merely dry boulders along stream in Picea-Abies forest, WGS84: 43.083N, 132.7E | TRH-B9515 |
| *Scapania spitsbergensis* (Lindb.) Müll.Frib. | NCBI:537859  GBIF:8990537  OTT:133607 | Laine, T. (1966): Enontekiö Lapland, Enontekiö, Porojärvet, Ridnitsohka mountain, in alpine top area, between boulders, 1250m above sea level | ZT-026-1966 |
| *Scapania subalpina* (Nees ex Lindenb.) Dumort. | NCBI:248359  GBIF:4277176  OTT:831943 | Hürlimann (1947) | Z-000129362 |
|  |  | Hangartner, R. (2008): GR Medel (Lucmagn), ob Masauna, 710.60/170.65, 1720m, Felsen feuchter Glimmerschiefer | ZT-101909-159278 |
|  |  | Hürlimann, H. (1975): Nasser Fels zwischen Restaurant und Staumauer Zervreila (Vals GR), ca. 1850müM | Z-000129211 |
|  |  | Peters, K. (2020): Schweiz, auf Erde in Felsspalte, 46.08316087, 7.923246937, 2585m, +-5m | Peters-2020-23 |
| *Scapania tundrae* (Arnell) H.Buch | NCBI:537860  GBIF:8547629  OTT:707922 | Sørensen, T. (1933): Liverpool Land: Cape Hope, Lat 70°28'N, Long: 220°25'W | CP-CM30429 |
| *Scapania uliginosa* (Lindenb.) Dumort. | NCBI:537861  GBIF:8368371  OTT:707920 | Urmi, E. (1984): Schweiz, Kt. Uri, Erstfeld Oberstafel (Riedbarg), 691.18 / 183.94, ca. 1530 müM, saures Hangmoor, in Sphagnum-Polster | Urmi-2825 |
| *Scapania umbrosa* (Schrad.) Dumort. | NCBI:537862  GBIF:4277183  OTT:707926 | Urmi, E. (1985): Schweiz, Nidwalden, Oberdorf oberhalb Wolfboden, CH1903+/LV95: 671.00 / 199.00, ca. 1340 müM, Lichter Fichtenwald an steilem Hang, auf totem Holz | Urmi-3527 |
|  |  | Edwin Urmi, 26.8.1983, Schweiz, Kt. Bern, Lenk Iffigtal bei der Iffigenalp, 599.88 / 138.6, ca. 1620 müM, im Fichtenwald, auf totem Baumstumpf Urmi-2548 |  |
| *Scapania undulata* (L.) Dumort. | NCBI:215256  GBIF:2689218  OTT:812077 | Bertram, J. (1996): VS Ried-Mörel, Reservat Aletschwald, Mittlerer Aletschwald, 645.54/138.08, 1970m, kleines Bächlein in einem Tälchen mit Alnetum viridis-Bestand auf Steinen im Bach, z.T. submers bzw. überspült | Bertram-1898e |
|  |  | Hürlimann, H. (1994): Fundort, Nasser schattiger Granitfels, Bondo GR im Bondasca-Tal bei Stauwehr-Brücke, 1080müM, 76370/13288 | Hürlimann-94211 |
| *Scapania verrucosa* Heeg | NCBI:537863  GBIF:8119128  OTT:707924 | Urmi, E. (1976): Italien, Piemonte, Prov. di Novara, Valstrona, am rechten Ufer des T. Strona westlich von Campello Monti, ret. ital.: fuso ovest 1440.8E / 5087.2 N, Bachböschung auf anstehendem Peridotit, ca 1320 müM | Urmi-1173 |
|  |  | P. Culmann, 22.8.1920 CM-30381 |  |
| *Scapania zemliae* S.W.Arnell | GBIF:7480007  OTT:7571345 | Holmen, K. (1958): From brooks below a glacier-tongue, Charcots Land: Lat 71°54'N, Long 29°W, Alt 850m | CP-CM30431 |

**Supplementary Table 2:** List of semantic identifiers used to annotate the image-enhancement performed in Adobe Camera-RAW.

| **Semantic identifier** | **Tag name** |
| --- | --- |
| {http://www.w3.org/1999/02/22-rdf-syntax-ns#} | about |
| {http://ns.adobe.com/xap/1.0/} | ModifyDate |
| {http://ns.adobe.com/xap/1.0/} | CreateDate |
| {http://ns.adobe.com/xap/1.0/} | MetadataDate |
| {http://ns.adobe.com/xap/1.0/} | Rating |
| {http://ns.adobe.com/tiff/1.0/} | Make |
| {http://ns.adobe.com/tiff/1.0/} | Model |
| {http://ns.adobe.com/tiff/1.0/} | Orientation |
| {http://ns.adobe.com/tiff/1.0/} | ImageWidth |
| {http://ns.adobe.com/tiff/1.0/} | ImageLength |
| {http://ns.adobe.com/exif/1.0/} | ExifVersion |
| {http://ns.adobe.com/exif/1.0/} | ExposureTime |
| {http://ns.adobe.com/exif/1.0/} | ShutterSpeedValue |
| {http://ns.adobe.com/exif/1.0/} | FNumber |
| {http://ns.adobe.com/exif/1.0/} | ApertureValue |
| {http://ns.adobe.com/exif/1.0/} | ExposureProgram |
| {http://ns.adobe.com/exif/1.0/} | SensitivityType |
| {http://ns.adobe.com/exif/1.0/} | RecommendedExposureIndex |
| {http://ns.adobe.com/exif/1.0/} | ExposureBiasValue |
| {http://ns.adobe.com/exif/1.0/} | MaxApertureValue |
| {http://ns.adobe.com/exif/1.0/} | MeteringMode |
| {http://ns.adobe.com/exif/1.0/} | FocalLength |
| {http://ns.adobe.com/exif/1.0/} | CustomRendered |
| {http://ns.adobe.com/exif/1.0/} | ExposureMode |
| {http://ns.adobe.com/exif/1.0/} | WhiteBalance |
| {http://ns.adobe.com/exif/1.0/} | SceneCaptureType |
| {http://ns.adobe.com/exif/1.0/} | FocalPlaneXResolution |
| {http://ns.adobe.com/exif/1.0/} | FocalPlaneYResolution |
| {http://ns.adobe.com/exif/1.0/} | FocalPlaneResolutionUnit |
| {http://ns.adobe.com/exif/1.0/} | DateTimeOriginal |
| {http://ns.adobe.com/exif/1.0/} | PixelXDimension |
| {http://ns.adobe.com/exif/1.0/} | PixelYDimension |
| {http://purl.org/dc/elements/1.1/} | format |
| {http://ns.adobe.com/exif/1.0/aux/} | SerialNumber |
| {http://ns.adobe.com/exif/1.0/aux/} | LensInfo |
| {http://ns.adobe.com/exif/1.0/aux/} | Lens |
| {http://ns.adobe.com/exif/1.0/aux/} | LensID |
| {http://ns.adobe.com/exif/1.0/aux/} | LensSerialNumber |
| {http://ns.adobe.com/exif/1.0/aux/} | ImageNumber |
| {http://ns.adobe.com/exif/1.0/aux/} | ApproximateFocusDistance |
| {http://ns.adobe.com/exif/1.0/aux/} | FlashCompensation |
| {http://ns.adobe.com/exif/1.0/aux/} | Firmware |
| {http://cipa.jp/exif/1.0/} | LensModel |
| {http://ns.adobe.com/photoshop/1.0/} | DateCreated |
| {http://ns.adobe.com/photoshop/1.0/} | SidecarForExtension |
| {http://ns.adobe.com/photoshop/1.0/} | EmbeddedXMPDigest |
| {http://ns.adobe.com/xap/1.0/mm/} | DocumentID |
| {http://ns.adobe.com/xap/1.0/mm/} | PreservedFileName |
| {http://ns.adobe.com/xap/1.0/mm/} | OriginalDocumentID |
| {http://ns.adobe.com/xap/1.0/mm/} | InstanceID |
| {http://ns.adobe.com/camera-raw-settings/1.0/} | Version |
| {http://ns.adobe.com/camera-raw-settings/1.0/} | ProcessVersion |
| {http://ns.adobe.com/camera-raw-settings/1.0/} | WhiteBalance |
| {http://ns.adobe.com/camera-raw-settings/1.0/} | Temperature |
| {http://ns.adobe.com/camera-raw-settings/1.0/} | Tint |
| {http://ns.adobe.com/camera-raw-settings/1.0/} | Sharpness |
| {http://ns.adobe.com/camera-raw-settings/1.0/} | LuminanceSmoothing |
| {http://ns.adobe.com/camera-raw-settings/1.0/} | ColorNoiseReduction |
| {http://ns.adobe.com/camera-raw-settings/1.0/} | VignetteAmount |
| {http://ns.adobe.com/camera-raw-settings/1.0/} | ShadowTint |
| {http://ns.adobe.com/camera-raw-settings/1.0/} | RedHue |
| {http://ns.adobe.com/camera-raw-settings/1.0/} | RedSaturation |
| {http://ns.adobe.com/camera-raw-settings/1.0/} | GreenHue |
| {http://ns.adobe.com/camera-raw-settings/1.0/} | GreenSaturation |
| {http://ns.adobe.com/camera-raw-settings/1.0/} | BlueHue |
| {http://ns.adobe.com/camera-raw-settings/1.0/} | BlueSaturation |
| {http://ns.adobe.com/camera-raw-settings/1.0/} | GrayMixerRed |
| {http://ns.adobe.com/camera-raw-settings/1.0/} | GrayMixerOrange |
| {http://ns.adobe.com/camera-raw-settings/1.0/} | GrayMixerYellow |
| {http://ns.adobe.com/camera-raw-settings/1.0/} | GrayMixerGreen |
| {http://ns.adobe.com/camera-raw-settings/1.0/} | GrayMixerAqua |
| {http://ns.adobe.com/camera-raw-settings/1.0/} | GrayMixerBlue |
| {http://ns.adobe.com/camera-raw-settings/1.0/} | GrayMixerPurple |
| {http://ns.adobe.com/camera-raw-settings/1.0/} | GrayMixerMagenta |
| {http://ns.adobe.com/camera-raw-settings/1.0/} | SplitToningShadowHue |
| {http://ns.adobe.com/camera-raw-settings/1.0/} | SplitToningShadowSaturation |
| {http://ns.adobe.com/camera-raw-settings/1.0/} | SplitToningHighlightHue |
| {http://ns.adobe.com/camera-raw-settings/1.0/} | SplitToningHighlightSaturation |
| {http://ns.adobe.com/camera-raw-settings/1.0/} | SplitToningBalance |
| {http://ns.adobe.com/camera-raw-settings/1.0/} | ParametricShadows |
| {http://ns.adobe.com/camera-raw-settings/1.0/} | ParametricDarks |
| {http://ns.adobe.com/camera-raw-settings/1.0/} | ParametricLights |
| {http://ns.adobe.com/camera-raw-settings/1.0/} | ParametricHighlights |
| {http://ns.adobe.com/camera-raw-settings/1.0/} | ParametricShadowSplit |
| {http://ns.adobe.com/camera-raw-settings/1.0/} | ParametricMidtoneSplit |
| {http://ns.adobe.com/camera-raw-settings/1.0/} | ParametricHighlightSplit |
| {http://ns.adobe.com/camera-raw-settings/1.0/} | SharpenRadius |
| {http://ns.adobe.com/camera-raw-settings/1.0/} | SharpenDetail |
| {http://ns.adobe.com/camera-raw-settings/1.0/} | SharpenEdgeMasking |
| {http://ns.adobe.com/camera-raw-settings/1.0/} | PostCropVignetteAmount |
| {http://ns.adobe.com/camera-raw-settings/1.0/} | GrainAmount |
| {http://ns.adobe.com/camera-raw-settings/1.0/} | ColorNoiseReductionDetail |
| {http://ns.adobe.com/camera-raw-settings/1.0/} | ColorNoiseReductionSmoothness |
| {http://ns.adobe.com/camera-raw-settings/1.0/} | LensProfileEnable |
| {http://ns.adobe.com/camera-raw-settings/1.0/} | LensManualDistortionAmount |
| {http://ns.adobe.com/camera-raw-settings/1.0/} | PerspectiveVertical |
| {http://ns.adobe.com/camera-raw-settings/1.0/} | PerspectiveHorizontal |
| {http://ns.adobe.com/camera-raw-settings/1.0/} | PerspectiveRotate |
| {http://ns.adobe.com/camera-raw-settings/1.0/} | PerspectiveScale |
| {http://ns.adobe.com/camera-raw-settings/1.0/} | PerspectiveAspect |
| {http://ns.adobe.com/camera-raw-settings/1.0/} | PerspectiveUpright |
| {http://ns.adobe.com/camera-raw-settings/1.0/} | PerspectiveX |
| {http://ns.adobe.com/camera-raw-settings/1.0/} | PerspectiveY |
| {http://ns.adobe.com/camera-raw-settings/1.0/} | AutoLateralCA |
| {http://ns.adobe.com/camera-raw-settings/1.0/} | Exposure2012 |
| {http://ns.adobe.com/camera-raw-settings/1.0/} | Contrast2012 |
| {http://ns.adobe.com/camera-raw-settings/1.0/} | Highlights2012 |
| {http://ns.adobe.com/camera-raw-settings/1.0/} | Shadows2012 |
| {http://ns.adobe.com/camera-raw-settings/1.0/} | Whites2012 |
| {http://ns.adobe.com/camera-raw-settings/1.0/} | Blacks2012 |
| {http://ns.adobe.com/camera-raw-settings/1.0/} | Clarity2012 |
| {http://ns.adobe.com/camera-raw-settings/1.0/} | Dehaze |
| {http://ns.adobe.com/camera-raw-settings/1.0/} | Texture |
| {http://ns.adobe.com/camera-raw-settings/1.0/} | ToneMapStrength |
| {http://ns.adobe.com/camera-raw-settings/1.0/} | ConvertToGrayscale |
| {http://ns.adobe.com/camera-raw-settings/1.0/} | OverrideLookVignette |
| {http://ns.adobe.com/camera-raw-settings/1.0/} | ToneCurveName |
| {http://ns.adobe.com/camera-raw-settings/1.0/} | ToneCurveName2012 |
| {http://ns.adobe.com/camera-raw-settings/1.0/} | CameraProfile |
| {http://ns.adobe.com/camera-raw-settings/1.0/} | CameraProfileDigest |
| {http://ns.adobe.com/camera-raw-settings/1.0/} | LensProfileSetup |
| {http://ns.adobe.com/camera-raw-settings/1.0/} | LensProfileName |
| {http://ns.adobe.com/camera-raw-settings/1.0/} | LensProfileFilename |
| {http://ns.adobe.com/camera-raw-settings/1.0/} | LensProfileDigest |
| {http://ns.adobe.com/camera-raw-settings/1.0/} | LensProfileDistortionScale |
| {http://ns.adobe.com/camera-raw-settings/1.0/} | LensProfileChromaticAberrationScale |
| {http://ns.adobe.com/camera-raw-settings/1.0/} | LensProfileVignettingScale |
| {http://ns.adobe.com/camera-raw-settings/1.0/} | UprightVersion |
| {http://ns.adobe.com/camera-raw-settings/1.0/} | UprightCenterMode |
| {http://ns.adobe.com/camera-raw-settings/1.0/} | UprightCenterNormX |
| {http://ns.adobe.com/camera-raw-settings/1.0/} | UprightCenterNormY |
| {http://ns.adobe.com/camera-raw-settings/1.0/} | UprightFocalMode |
| {http://ns.adobe.com/camera-raw-settings/1.0/} | UprightFocalLength35mm |
| {http://ns.adobe.com/camera-raw-settings/1.0/} | UprightPreview |
| {http://ns.adobe.com/camera-raw-settings/1.0/} | UprightDependentDigest |
| {http://ns.adobe.com/camera-raw-settings/1.0/} | UprightTransformCount |
| {http://ns.adobe.com/camera-raw-settings/1.0/} | UprightTransform_0 |
| {http://ns.adobe.com/camera-raw-settings/1.0/} | UprightTransform_1 |
| {http://ns.adobe.com/camera-raw-settings/1.0/} | UprightTransform_2 |
| {http://ns.adobe.com/camera-raw-settings/1.0/} | UprightTransform_3 |
| {http://ns.adobe.com/camera-raw-settings/1.0/} | UprightTransform_4 |
| {http://ns.adobe.com/camera-raw-settings/1.0/} | UprightTransform_5 |
| {http://ns.adobe.com/camera-raw-settings/1.0/} | UprightFourSegmentsCount |
| {http://ns.adobe.com/camera-raw-settings/1.0/} | ToggleStyleDigest |
| {http://ns.adobe.com/camera-raw-settings/1.0/} | ToggleStyleAmount |
| {http://ns.adobe.com/camera-raw-settings/1.0/} | HasSettings |
| {http://ns.adobe.com/camera-raw-settings/1.0/} | CropTop |
| {http://ns.adobe.com/camera-raw-settings/1.0/} | CropLeft |
| {http://ns.adobe.com/camera-raw-settings/1.0/} | CropBottom |
| {http://ns.adobe.com/camera-raw-settings/1.0/} | CropRight |
| {http://ns.adobe.com/camera-raw-settings/1.0/} | CropAngle |
| {http://ns.adobe.com/camera-raw-settings/1.0/} | CropConstrainToWarp |
| {http://ns.adobe.com/camera-raw-settings/1.0/} | HasCrop |
| {http://ns.adobe.com/camera-raw-settings/1.0/} | AlreadyApplied |
| {http://ns.adobe.com/camera-raw-settings/1.0/} | RawFileName |
| {http://ns.adobe.com/camera-raw-settings/1.0/} | Saturation |
| {http://ns.adobe.com/camera-raw-settings/1.0/} | Vibrance |
| {http://ns.adobe.com/camera-raw-settings/1.0/} | HueAdjustmentRed |
| {http://ns.adobe.com/camera-raw-settings/1.0/} | HueAdjustmentOrange |
| {http://ns.adobe.com/camera-raw-settings/1.0/} | HueAdjustmentYellow |
| {http://ns.adobe.com/camera-raw-settings/1.0/} | HueAdjustmentGreen |
| {http://ns.adobe.com/camera-raw-settings/1.0/} | HueAdjustmentAqua |
| {http://ns.adobe.com/camera-raw-settings/1.0/} | HueAdjustmentBlue |
| {http://ns.adobe.com/camera-raw-settings/1.0/} | HueAdjustmentPurple |
| {http://ns.adobe.com/camera-raw-settings/1.0/} | HueAdjustmentMagenta |
| {http://ns.adobe.com/camera-raw-settings/1.0/} | SaturationAdjustmentRed |
| {http://ns.adobe.com/camera-raw-settings/1.0/} | SaturationAdjustmentOrange |
| {http://ns.adobe.com/camera-raw-settings/1.0/} | SaturationAdjustmentYellow |
| {http://ns.adobe.com/camera-raw-settings/1.0/} | SaturationAdjustmentGreen |
| {http://ns.adobe.com/camera-raw-settings/1.0/} | SaturationAdjustmentAqua |
| {http://ns.adobe.com/camera-raw-settings/1.0/} | SaturationAdjustmentBlue |
| {http://ns.adobe.com/camera-raw-settings/1.0/} | SaturationAdjustmentPurple |
| {http://ns.adobe.com/camera-raw-settings/1.0/} | SaturationAdjustmentMagenta |
| {http://ns.adobe.com/camera-raw-settings/1.0/} | LuminanceAdjustmentRed |
| {http://ns.adobe.com/camera-raw-settings/1.0/} | LuminanceAdjustmentOrange |
| {http://ns.adobe.com/camera-raw-settings/1.0/} | LuminanceAdjustmentYellow |
| {http://ns.adobe.com/camera-raw-settings/1.0/} | LuminanceAdjustmentGreen |
| {http://ns.adobe.com/camera-raw-settings/1.0/} | LuminanceAdjustmentAqua |
| {http://ns.adobe.com/camera-raw-settings/1.0/} | LuminanceAdjustmentBlue |
| {http://ns.adobe.com/camera-raw-settings/1.0/} | LuminanceAdjustmentPurple |
| {http://ns.adobe.com/camera-raw-settings/1.0/} | LuminanceAdjustmentMagenta |
| {http://ns.adobe.com/camera-raw-settings/1.0/} | DefringePurpleAmount |
| {http://ns.adobe.com/camera-raw-settings/1.0/} | DefringePurpleHueLo |
| {http://ns.adobe.com/camera-raw-settings/1.0/} | DefringePurpleHueHi |
| {http://ns.adobe.com/camera-raw-settings/1.0/} | DefringeGreenAmount |
| {http://ns.adobe.com/camera-raw-settings/1.0/} | DefringeGreenHueLo |
| {http://ns.adobe.com/camera-raw-settings/1.0/} | DefringeGreenHueHi |
| {http://ns.adobe.com/xap/1.0/} | Label |
| {http://ns.adobe.com/camera-raw-settings/1.0/} | VignetteMidpoint |
